# Multiple mechanisms underlie reduced potassium conductance in the p.T1019PfsX38 variant of hERG

**DOI:** 10.14814/phy2.15341

**Published:** 2022-07-19

**Authors:** Majid K. Al Salmani, Rezvan Tavakoli, Wajid Zaman, Ahmed Al Harrasi

**Affiliations:** ^1^ 120654 Natural and Medical Sciences Research Center University of Nizwa Nizwa Oman

**Keywords:** arrhythmia, hERG, long QT syndrome, potassium channels

## Abstract

Long QT syndrome type II (LQT2) is caused by loss‐of‐function mutations in the hERG K^+^ channel, leading to increased incidence of cardiac arrest and sudden death. Many genetic variants have been reported in the hERG gene with various consequences on channel expression, permeation, and gating. Only a small number of LQT2 causing variants has been characterized to define the underlying pathophysiological causes of the disease. We sought to determine the characteristics of the frameshift variant p.Thr1019ProfsX38 (T1019PfsX38) which affects the C‐terminus of the protein. This mutation was identified in an extended Omani family of LQT2. It replaces the last 140 amino acids of hERG with 37 unique amino acids. T1019 is positioned at a distinguished region of the C‐terminal tail of hERG, as predicted from the deep learning system AlphaFold v2.0. We employed the whole‐cell configuration of the patch‐clamp technique to study wild‐type and mutant channels that were transiently expressed in human embryonic kidney 293 (HEK293) cells. Depolarizing voltages elicited slowly deactivating tail currents that appeared upon repolarization of cells that express either wild‐type‐ or T1019PfsX38‐hERG. There were no differences in the voltage and time dependencies of activation between the two variants. However, the rates of hERG channel deactivation at hyperpolarizing potentials were accelerated by T1019PfsX38. In addition, the voltage dependence of inactivation of T1019PfsX38‐hERG was shifted by 20 mV in the negative direction when compared with wild‐type hERG. The rates of channel inactivation were increased in the mutant channel variant. Next, we employed a step‐ramp protocol to mimic membrane repolarization by the cardiac action potential. The amplitudes of outward currents and their integrals were reduced in the mutant variant when compared with the wild‐type variant during repolarization. Thus, changes in the gating dynamics of hERG by the T1019PfsX38 variant contribute to the pathology seen in affected LQT2 patients.

## Graphical abstract

Dynamic voltage clamp conditions reveal gating defects that reduce potassium conductance through the T1019PfsX38 variant of the hERG channel. This has important implications for the future development of personalized therapies for long QT in affected individuals.

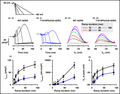

## 1 INTRODUCTION

The human Ether‐à‐go‐go‐Related Gene (hERG, K_V_11.1, *KCNH2*) is a voltage‐dependent K^+^ channel highly expressed in cardiac myocytes. hERG is a multidomain protein of which two major isoforms have been identified (hERG 1a and 1b). hERG assembles into a tetrameric structure to perform its function at the plasma membrane. It plays a central physiological role by mediating fast potassium conductance near the end of the cardiac action potential (AP). Each subunit of the tetrameric structure contains intracellular N‐ and C‐terminals, and six transmembrane helices (S1–S6). The ion selectivity and voltage sensitivity of hERG are regulated by the transmembrane helices (Warmke & Ganetzky, 1994). The N‐terminus contains a globular eag domain which differs in structure between hERG 1a and 1b isoforms. The C‐terminus constitutes a cyclic nucleotide‐binding homology domain (cNBHD) and a long C‐tail (Warmke & Ganetzky, 1994). Several studies have reported the involvement of the N‐ and C‐termini in channel gating, but their full contribution to channel function is not fully understood.

hERG is a major component of the delayed rectifier potassium current (I_Kr_) which terminates the cardiac AP (Sanguinetti et al., 1995). Due to its fast inactivation and slow deactivation rates, hERG activity remains minimal before the end of the plateau phase of the cardiac AP. During the repolarization phase, hERG rapidly recovers from inactivation leading to increased outward K^+^ currents. This brings the membrane potential back to its resting state. Hence, hERG plays a major role in determining the length of the cardiac AP. The structural basis for this unique gating behavior is not fully understood. Previous pharmacological investigations and site‐directed mutagenesis studies have provided wealth of information about key structural sites which regulate the transition between the different gating states of hERG (Vandenberg et al., 2012).

Loss of function mutations in hERG cause long QT syndrome type II (LQT2) (Curran et al., 1995), whereas gain of function mutations cause short QT syndrome type I (SQT1) (Brugada et al., 2004; Delisle et al., 2004). There are potentially >1000 reported variations in the hERG gene (Leiden Open Variation Database, https://database.lovd.nl/shared/genes). The disease causing variants could be put into 4 classes according to their underlying molecular pathologies (Anderson et al., 2006). These classes are: Class 1, mutations that impair protein synthesis; Class 2: mutations that impair protein folding, assembly, and trafficking to the cell surface; Class 3, mutations that modulate channel gating properties; and Class 4, mutations that affect ion conductance through the pore domain. Only a small fraction of the known hERG variants has been functionally characterized.

The T1019PfsX38 (c.3054delC) mutation in the hERG channel was reported in an extended family of LQT2 from Oman (Al Senaidi et al., 2014). It caused a prolonged corrected QT (QTc) interval (>570 ms) in a 5 years old girl who was homozygote for this mutation. Heterozygote individuals had borderline QTc intervals (Al Senaidi et al., 2014). This mutation affects the C‐tail of hERG and results in a shift in the open reading frame. In this study, we performed investigations to understand the functional consequences of the frameshift mutation T1019PfsX38 on hERG channel activity at the plasma membrane.

## 2 MATERIALS AND METHODS

### 2.1 Site directed mutagenesis

We modified pcDNA3.0 plasmid that contains WT‐hERG cDNA to insert the T1019PfsX38 mutation. The plasmid was originally from Dr. John Mitcheson, University of Leicester. We performed site‐directed mutagenesis using QuikChange Lightning Site‐Directed Mutagenesis Kit (Catalog # 210518, Agilent Technologies, Santa Clara, CA, USA) following manufacturer instructions. The polymerase chain reaction (PCR) primers were 5ʹ‐gccccgccccacccccagcc‐3ʹ and 5ʹ‐ggctgggggtggggcggggc‐3ʹ (Eurofins Genomics, Luxembourg). Briefly, we performed PCR using 100 ng of template plasmid and 125 ng of each primer in a 25 uL final volume. The PCR programme included an initial denaturation at 95°C (2 min), and 18 cycles of 95°C (20 s), 60°C (10 s), and 68°C (5 min). The cycles were followed by a final elongation for 5 min at 68°C and incubation at 4°C thereafter. Then, we added 1 µL of DpnI Enzyme to the PCR mixture for 10 min at 37°C to digest parental DNA. Next, we transformed 100 µL of XL10‐Gold Ultracompetent Cells using 7 µL of the PCR product. After 1 h of incubation at 37°C with LB‐broth media, we plated the cells on LB‐ampicillin (100 µg/mL) selective agar plates overnight at 37°C. Finally, we extracted plasmid DNA from the appearing bacterial colonies and confirmed the presence of the mutation by Sanger sequencing (Eurofins Genomics, Cologne, Germany).

### 2.2 Mammalian cell culture and transfection

For patch‐clamp recording, we used human embryonic kidney 293 (HEK‐293) cell line (passage <40) (ECACC, MERCK, Catalog # 85120602) grown in Dulbecco's Modified Eagle Medium (DMEM) high glucose (Sigma‐Aldrich, St. Louis, MO, USA). Cells were maintained in 25 cm^2^ flasks or 6‐well plates at 37°C (5% CO_2_). Before transfection, the cells were washed with phosphate‐buffered saline (PBS) for 1 min then treated with 0.25% trypsine‐EDTA (Sigma Aldrich, St. Louis, MO, USA). The cells were then seeded onto 35 mm petri dishes (Carl Roth, Karlsruhe, Germany) containing 22 mm glass coverslips (Carl Roth, Karlsruhe, Germany). After 24–48 h of incubation, we transfected these cells using a mixture of 2.5 μg of hERG plasmid DNA and 1.5 μg of enhanced green fluorescent protein N1 (EGFP‐N1) and Lipofectamine 2000 (Thermofisher Scientific, Waltham, MA, USA). We used the cells for experiments 24–96 h after transfection.

### 2.3 Electrophysiology solutions and microelectrode preparation

The extracellular solution contained in mM: 140 NaCl, 4 KCl, 2.5 CaCl_2_, 1 MgCl_2_, 10 D‐glucose, and 5 HEPES, adjusted to pH 7.45 with NaOH. The intracellular solution contained in mM: 130 KCl, 5 MgCl_2_, 5 EGTA, 5 Na_2_ATP, and 10 HEPES, adjusted to pH 7.2 with KOH. Fresh Na_2_ATP from 200 mM stock was added to the intracellular solution before every experiment and stored on ice, pH was adjusted accordingly. All chemical compounds used in the solutions were from Carl Roth (Karlsruhe, Germany).

Glass pipettes were made freshly before each experiment using premium thin wall borosilicate capillary glass with filament (model G150TF‐4, Warner Instruments, USA). The pipettes were pulled using PC‐100 puller (Narishige, Tokyo, Japan). All pipettes had resistance of ~3.6 MΩ when put in the bath solution.

### 2.4 Patch‐clamp setup and data acquisition

The patch‐clamp setup was composed of a recoding chamber (Model RC‐24E, Warner instruments, Hamden, CT, USA) mounted on an inverted phase‐contrast microscope (Model IX73, Olympus Corporation, Tokyo, Japan) that was equipped with fluorescence visualization. The bath solution was applied by pump‐assisted perfusion using a syringe. Reference electrodes were made using bent glass capillaries that were mounted onto the recording chamber and contained 2.5 M KCl in 1% agarose. To isolate mechanical vibrations, the microscope was put on CleanBench laboratory table equipped with a Faraday cage (Technical Manufacturing Corporation, Peabody, MA, USA). The currents were amplified using a PC‐505B whole cell/patch‐clamp amplifier (Warner instruments, Hamden, CT, USA) or Axopatch 200b (Molecular Devices, San Jose, CA, USA), and acquired using Digidata 1322A/1550B and pClamp 9.2/11.2 software (Molecular Devices, San Jose, CA, USA). The signals were low‐pass filtered at 10 kHz and sampled at 20 kHz. The amplifier headstage was mounted on PatchStar MicroManipulator Controller (Scientifica Limited, East Sussex, UK). All voltage‐clamp protocols are described in the results section. All recordings were acquired at room temperature (24–26°C).

### 2.5 Patch‐clamp procedure

First, we transferred the cells onto the recoding chamber and mounted the recording pipette onto the pipette holder. Then, we applied gentle positive pressure to the back of the pipette and submerged its tip into the bath solution. The resistance was monitored on the computer screen. We identified successfully transfected cells by EGFP fluorescence and targeted them. After correcting for pipette offset, we gently lowered the pipette and placed it onto the surface of the target cell. Then, we applied gentle negative pressure to form a high‐resistance seal. When this was achieved, we switched the amplifier to the whole‐cell configuration and ruptured the seal by gentle suction. Then, we immediately held the cell at −80 mV and compensated capacitive transients using the given controls. The series resistance was compensated by ~80%.

### 2.6 Data analysis, statistics, and software

**FIGURE 1:**
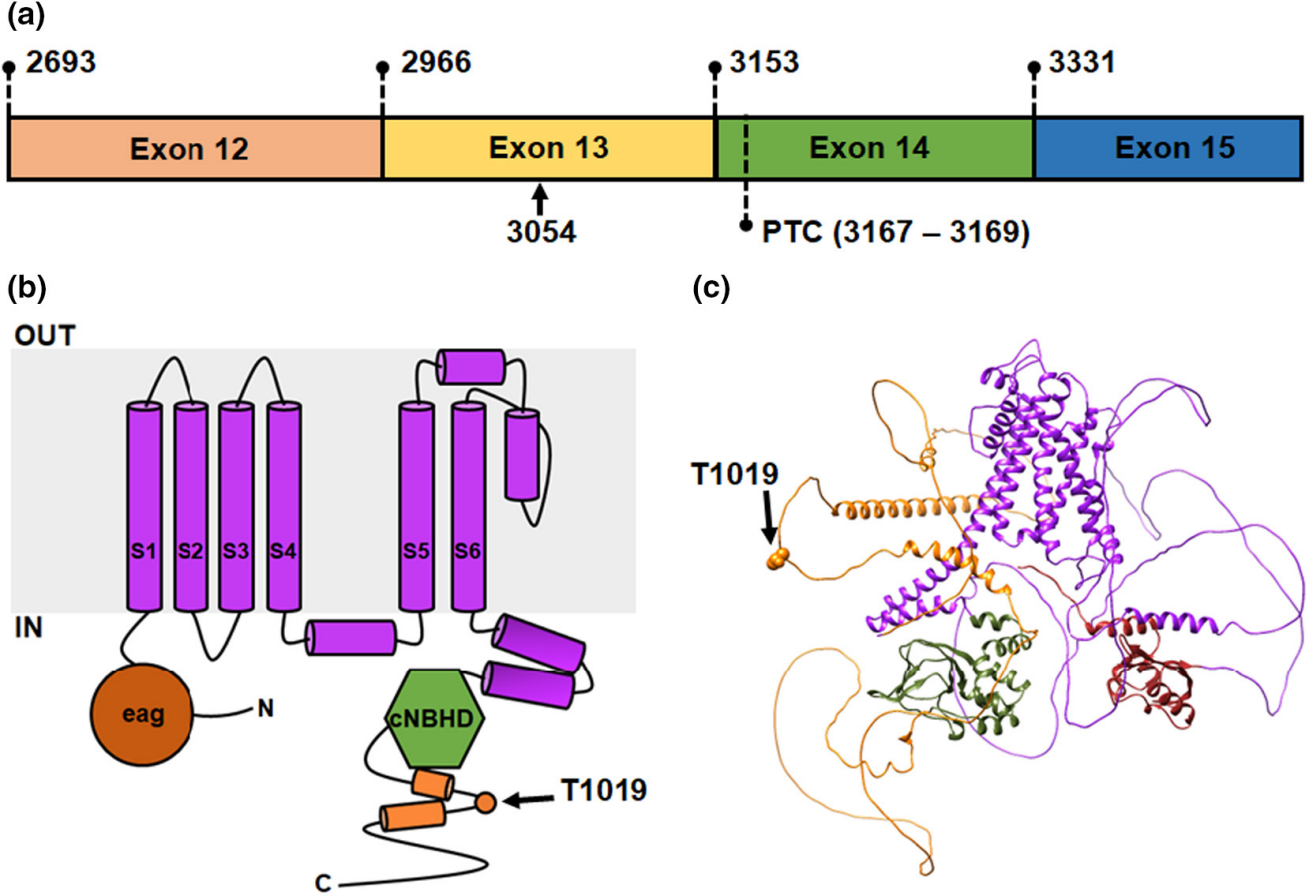
Gene and protein location of T1019PfsX38. (a) The location of the c.3054delC (p.T1019PfsX38) within the cDNA of the KCNH2 gene. The numbers and labels indicate exons start sites (top), and positions of the deletion (3054) and the resulting PTC. (b) The position of T1019 in the linear structure of a single hERG channel subunit. The structure shows six transmembrane segments (S1–S6), the eag domain and the cNBHD. (c) The location of T1019 in the structure of a single hERG subunit as predicted by AlphaFold v2.0 (Jumper et al., 2021). The structural domains in c were color‐coded as in b. Other details as in Introduction, Materials and methods, and Results sections.

All patch‐clamp data and analyses were obtained and fitted using either Clampfit 9.2 or Clampfit 11.2 (batch analysis). We fitted tail currents with a Boltzmann equation, I = (I_max_/(1 + exp ((V_mid_ – V_m_)/k))) + C to determine the half‐maximum voltage (V_mid_) and slope factor (k) of activation and inactivation. The rates of tail currents recovery and relaxation were obtained by standard fit using 1‐ and 2‐component exponential equations, respectively. Statistical analyses were performed using SigmaPlot 14.0 (Systat Software Inc, San Jose, CA, USA). Data was tested for normality using Shapiro–Wilk test before performing statistical analyses. Any statistical difference was considered significant if *n* ≥ 5 and *p* < 0.05 using two‐tailed Student’s *t*‐test. As indicated in Figure 9 legend, we used Mann‐Whitney rank sum test for statistical comparison at a few data points where statistical normal distribution of data was not met. Unless otherwise indicated, data are mean ± SEM, n is the number of cells. Figures were drawn using SigmaPlot 14.0 (Systat Software Inc, San Jose, CA, USA). The 3D model of hERG protein structure (Figure 1) was visualized and plotted using UCSF Chimera 1.15 (Pettersen et al., 2004) and Microsoft PowerPoint 2016 (Microsoft Corporation, WA, USA).

## 3 RESULTS

### 3.1 T1019PfsX38 in the hERG gene and protein

T1019PfsX38 (c.3054delC) is located in the distal part of the hERG gene. Figure 1 shows the position of this variant within the cDNA and protein of hERG. The nucleotide deletion occurs in the centre of exon 13 causing a shift in the open reading frame (Figure 1a). As a result, a premature termination codon (PTC) forms 16 nucleotide into exon 14. The C‐tail starts at amino acid position of ~857 and ends at 1159. T1019PfsX38 modifies the long C‐tail of hERG, rendering all of the transmembrane segments and the cytoplasmic globular domains unchanged (Figure 1b). The published cryo‐electron microscopy (cryo‐EM) structures of hERG including Wang and MacKinnon (2017) lacked the C‐tail. Thus, we utilized the structure predicted by AlphaFold v2.0, an artificial intelligence program developed by Google's DeepMind learning system (Jumper et al., 2021). AlphaFold v2.0 predicts that the majority of the C‐tail is a largely disordered region, except for two α‐helical segments 989–1003 and 1035–1073 (Figure 1b,c). T1019 is situated in the middle of the disordered region between the two predicted α‐helical segments of the C‐tail. Thus, the frameshift starting at T1019 occurs in close proximity to the first α‐helix of the C‐tail and deletes the second α‐helix.

### 3.2 Densities of whole‐cell currents in transiently transfected HEK293 cells

**FIGURE 2:**
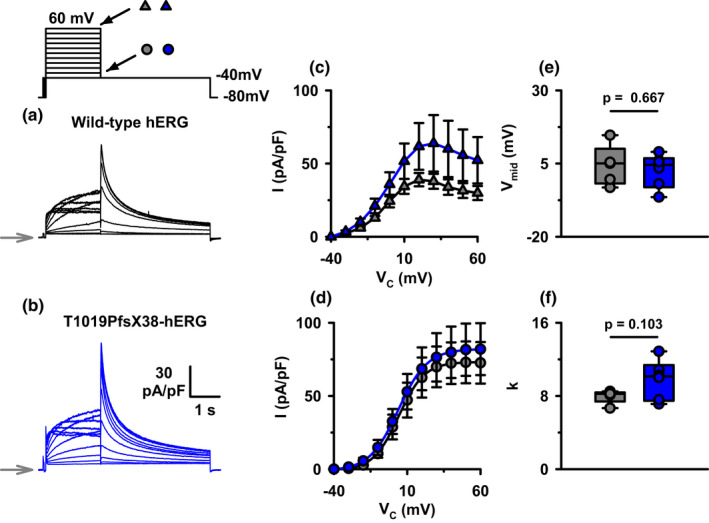
The current densities and the voltage dependence of activation of WT‐ and T1019PfsX38‐hERG channels. (a, b) Representative whole‐cell currents recorded from WT‐ (a) and T1019PfsX38‐hERG (b) channels expressed in HEK293 cells. The currents were elicited using the protocol shown above A (V_hold_ = −80 mV). The grey arrows mark the level where V_C_ was −80 mV. (c) Quantification of step currents measured as average current densities in the last 50 ms of the depolarizing test potentials. (d) Quantification of peak tail currents measured after stepping to −40 mV. The continuous lines in (d) are best fits to the average data using the Boltzmann equation. (e, f) Whisker plots of the V_mid_ and k values predicted by fitting the data in (d). Data in (c) and (d) are mean ± SEM; WT‐hERG, *n* = 5, grey circles and triangles; T1019PfsX38‐hERG, *n* = 6, blue circles and triangles. Other details as in Materials and Methods and Results sections.

To test whether the activity of T1019PfsX38‐hERG could be detected at the plasma membrane and to compare it with the activity of the wild‐type variant, we measured the densities of hERG currents in HEK293 cells that were transiently transfected with WT or mutant hERG cDNA using a standard patch clamp protocol. The activity of the hERG channel could be distinguished by the high degree of inactivation at positive potentials and fast recovery from inactivation upon repolarization. In addition, the tail currents of hERG have slow rates of deactivation. We used a modified protocol from El Harchi et al. (2018). First, the cells were depolarized briefly to −40 mV for 40 ms from a holding potential of −80 mV. Then we used 2 s depolarizing steps to activate hERG at potentials ranging from −40 to 60 mV in 10 mV increments. Then we repolarized the membrane to −40 mV for 4 s. Figure 2 shows example recordings and quantification of hERG channel activity in response to this voltage‐clamp protocol. Visual inspection of the recordings in Figure 2a,b indicates the presence of currents that increased upon depolarization. Subsequent repolarization to −40 mV elicited outward tail currents that were higher in amplitude than those elicited at most test potentials. The densities of currents detected at the end of the depolarizing steps were similar in both WT‐ and T1019PfsX38‐hERG expressing cells (Figure 2c) (*p* > 0.05). Moreover, there were no differences between the densities of the tail currents produced by WT‐ or T1019PfsX38‐hERG channels (Figure 2d) (*p* > 0.05).

### 3.3 The voltage dependence of activation

hERG channel activation is voltage dependent (Trudeau et al., 1995). The protocol presented in Figure 2 could be used to measure the voltage dependence of activation of hERG channels. There was strong inward rectification of currents at the end of the 2 s depolarizing steps (Figure 2a,b). Repolarization to −40 mV elicited rapid outward tail currents that decayed slowly within a few seconds. The current‐to‐voltage (IV) relationships presented in Figure 2c had sigmoidal shapes at potentials lower than 20 mV, and inwardly rectified at more positive potentials. WT‐ and T1019PfsX38‐hERG channels showed similar behaviors at all tested potentials (Figure 2c) (*p* > 0.05).

The inward rectification of K^+^ currents at positive potentials in Figure 2(c) is a result of hERG channel inactivation (Trudeau et al., 1995). Upon repolarization, many inactive channels reopen, allowing an indirect method to measure the relative extent of channel activation at the proceeding depolarizing steps. Peak tail currents (I_peak_) of WT‐ and T1019PfsX38‐hERG channels reached maximal values when depolarization was ≥40 mV (Figure 2d). To quantify the voltage dependence of activation, we fitted I_peak_ values using the Boltzmann function (Figure 2d). We did not observe significant differences in the V_mid_ and k of activation between WT‐ and T1019PfsX38‐hERG channels (Figure 2e,f) (*p* > 0.05). Thus, we can conclude that T1019PfsX38 does not change the voltage dependence of activation of hERG at steady‐state conditions.

### 3.4 The time dependence of activation at different membrane potentials

**FIGURE 3:**
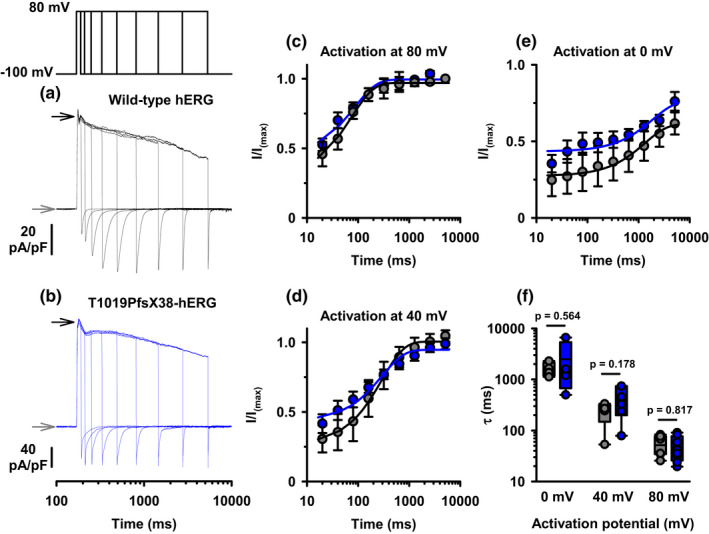
Kinetics of activation of WT‐ and T1019PfsX38‐hERG channels. (a, b) Example whole cell currents recorded from WT‐ (a) and T1019PfsX38‐hERG (b) channels expressed in HEK293 cells. The currents were elicited using the protocol shown above (a). (V_hold_ = −100 mV). The example recordings in (a) and (b) were from cells activated at 80 mV. For better visualization, the x‐axes in (a) and (b) were presented in logarithmic scales. The grey and black arrows mark the levels where V_C_ was −100 mV and 80 mV, respectively. (c–e) Quantification of the negative peak tail currents obtained after activating the channels at 80 mV (c), 40 mV (d) or 0 mV (e) and presented as fractions of the peak inward tail currents after depolarization to 80 mV for 5.12 s in the same cell. The continuous lines in (c–e) are best fits to the average data using an exponential function. (f) Whisker plots and individual data points of the time constants of activation of WT‐ and T1019PfsX38‐hERG channels. Data are mean ± SEM; WT‐hERG, grey circles/lines, *n* = 7–8 (c), *n* = 5–6 (d), *n* = 5 (e) and *n* = 4–7 (f); T1019PfsX38‐hERG, blue circles / lines, *n* = 7–9 (c), *n* = 6–7 (d), *n* = 6 (e) and *n* = 4–8 (f). Other details as in Materials and Methods, and Results sections.

The rate of channel activation is a factor that determines the conductance level of hERG at different stages of the cardiac AP. To quantify the activation kinetics at depolarizing potentials, we had to separate activation from inactivation by utilizing the process of fast recovery from inactivation of hERG upon repolarization. Figure 3 presents data that quantifies the extent of WT‐ and T1019PfsX38‐hERG channel activation at various time points. Briefly, HEK293 cells that transiently expressed WT‐ or T1019PfsX38‐hERG channels were depolarized to either 80, 40 or 0 mV for logarithmically increasing durations ranging from 20 to 5120 ms. Then, the cells were stepped to −100 mV to induce channel recovery from inactivation which enabled us to quantify the extent of hERG activation during the preceding step. Figure 3a,b show example recordings obtained using this protocol at 80 mV activation potential. Visual inspection of the recordings indicates that hERG currents were activated considerably within 20 ms when the membrane was depolarized to 80 mV in both WT‐ and T1019PfsX38‐hERG expressing cells. The currents increased in an exponential manner to a maximal value that was reached within less than 1 s. Figure 3c presents quantification of the currents that were activated at different time durations following depolarization to 80 mV. The currents in Figure 3c are presented as fractions of the currents obtained at the end of the longest activation step at 80 mV. The rates of channel activation was high for both WT‐ and T1019PfsX38‐hERG, and reached maximal values between 300 and 500 ms (Figure 3c). The two variants had similar patterns of activation at all tested activation potentials (Figure 3c‐e). At 40 mV, both variants required >1 s to be maximally activated (Figure 3d). The maximal activity could not be reached at 0 mV even after 5 s of depolarization (Figure 3e). We fitted the data from individual cells using an exponential function that quantified the rates of channel activation. Figure 3f presents individual data points and Whisker plots of activation τ at different membrane potentials. We did not observe significant differences in τ of activation between WT‐ and T1019PfsX38‐hERG at all tested potentials (Figure 3f). Thus, we conclude that the time dependence of activation of hERG was not modified by the T1019PfsX38 variant.

### 3.5 Recovery from inactivation and deactivation

**FIGURE 4:**
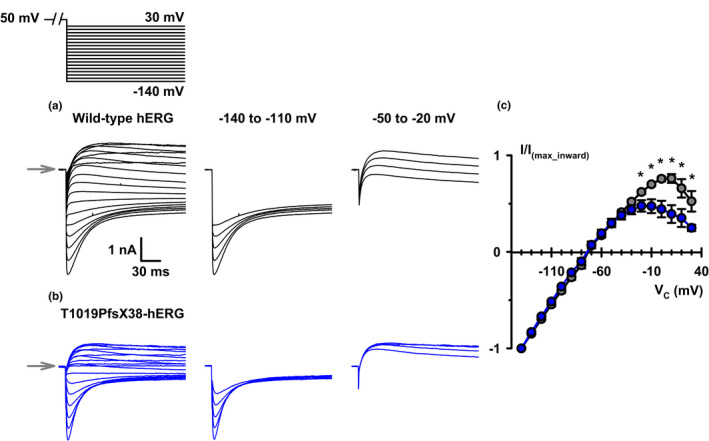
The voltage dependence of recovery from inactivation of WT‐ and T1019PfsX38‐hERG channels. (a, b) Representative whole cell currents recorded from WT‐ (a) and T1019PfsX38‐hERG (b) channels expressed in HEK293 cells. The currents were elicited using the protocol shown above A (V_hold_ = −80 mV). The grey arrows mark the level where V_C_ was 50 mV. For better representation, isolated segments from (a) and (b) at different test potentials are shown separately. (c) Quantification of peak current amplitudes measured after stepping to the test potential and normalized to the maximum inward current amplitudes. Data are mean ± SEM, *p < 0.05; WT‐hERG, *n* = 6, grey; T1019PfsX38‐hERG, *n* = 7, blue. Other details as in Materials and Methods and Results sections.

The tail currents that we observed in Figure 2 upon membrane repolarization developed rapidly and then deactivated slowly as the active channels returned to the closed state. To test whether T1019PfsX38 affects the behavior of channel recovery from inactivation or the process of deactivation, we studied the tail currents at different membrane repolarization potentials. Figure 4 shows the protocol that we utilized to elicit tail currents at potentials between −140 and 30 mV and representative current recordings from WT‐ and T1019PfsX38‐hERG expressing cells. Visual inspection of the example recordings in Figure 4a,b show that the tail currents at hyperpolarizing potentials reached a peak within a few milliseconds then decayed steeply in an exponential manner. Notably, the decay rates of the tail currents were slower at depolarizing potentials than at hyperpolarising potentials. Figure 4c shows quantification of tail currents normalized to the maximum inward tail currents in the same cells. The normalized tail currents of WT‐hERG had linear IV relationship at hyperpolarising potentials but inwardly rectified at potentials above –30 mV (Figure 4c). In contrast, the inward rectification of the tail currents of T1019PfsX38‐hERG started at more negative potentials and could already be observed at or above −50 mV in Figure 4c. As a result, the normalized peak tail current amplitudes of WT‐hERG in Figure 4c were higher than those of T1019PfsX38‐hERG at potentials ≥−30 mV. The tail currents of WT‐ and T1019PfsX38‐hERG peaked at 8.33 ± 4.01 mV (*n* = 6) and −15.71 ± 3.69 mV (*n* = 7), respectively (*p* = 0.001).

**FIGURE 5:**
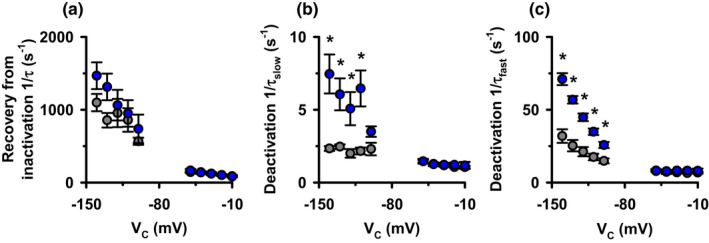
Quantification of the rate constants of WT‐ and T1019PfsX38‐hERG recovery from inactivation and deactivation. (a–c) Average values of the rate constants obtained by fitting the tail currents that were elicited upon repolarization from 50 mV (2 s) to different test potentials, as in Figure 4. The data were obtained by fitting >1.5 and >0.5 s of the currents that were observed at depolarized and hyperpolarized potentials, respectively. Data are mean ± SEM, *p < 0.05; WT‐hERG, grey circles; T1019PfsX38‐hERG, blue circles. Other details as in Figure 4, Table 1, Materials and Methods and Results sections.

To better understand the IV relationship of the elicited tail currents in Figure 4c, we measured the rates of transition between the different apparent gating configurations. We fitted the tail currents with exponential components to measure the rate constants of recovery from inactivation and deactivation. The recovery of currents from inactivation was monophasic while the deactivation of currents was biphasic. Figure 5 shows quantification of the rate constants measured by fitting the inward (−140 to −100 mV) and the outward (−50 to −10 mV) tail currents using three exponential components. The rates of channel recovery from inactivation were faster at hyperpolarizing potentials than depolarizing potentials for both variants (Figure 5 present). There were no apparent differences in the rates of recovery from inactivation between WT‐ and T1019PfsX38‐hERG at all tested potentials (Figure 5a). Channel deactivation had slow and fast decay components, τ_slow_ and τ_fast_ (Figure 5b,c). At hyperpolarizing potentials (−140 to 110 mV) the rates of deactivation were a few fold faster for T1019PfsX38‐hERG when compared with WT‐hERG (Figure 5b,c) (*p* < 0.025). In contrast, at depolarizing potentials, the deactivation rates were not different between the two variants (Figure 5b,c). Table 1 shows average values of fractional amplitudes of the fitted deactivation components. The relative contribution of the fast and the slow decay components were not affected by T1019PfsX38 at all tested potentials (Table 1).

**TABLE 1:** Values of fractional amplitudes of exponential deactivation components. The values were obtained by fitting the decay curves of the currents shown in Figure 4 using two exponential component fits. Values are mean ± SD. Other details as in Figures 4 and 5, Materials and methods and Results sections

	WT				T1019PfsX38			
V_C_ (mV)	a_slow_	a_fast_	SD	*n*	a_slow_	a_fast_	SD	*n*
−140	0.14	0.86	0.02	6	0.11	0.89	0.04	6
−130	0.12	0.88	0.02	6	0.12	0.88	0.04	6
−120	0.13	0.87	0.04	6	0.13	0.87	0.04	6
−110	0.18	0.82	0.18	6	0.17	0.83	0.08	5
−100	0.17	0.83	0.09	5	0.11	0.89	0.06	5
−50	0.57	0.43	0.07	4	0.58	0.42	0.09	7
−40	0.62	0.38	0.07	5	0.63	0.37	0.06	7
−30	0.68	0.32	0.12	6	0.67	0.33	0.07	6
−20	0.66	0.34	0.13	6	0.65	0.35	0.10	5
−10	0.65	0.35	0.10	6	0.60	0.40	0.05	4

**FIGURE 6:**
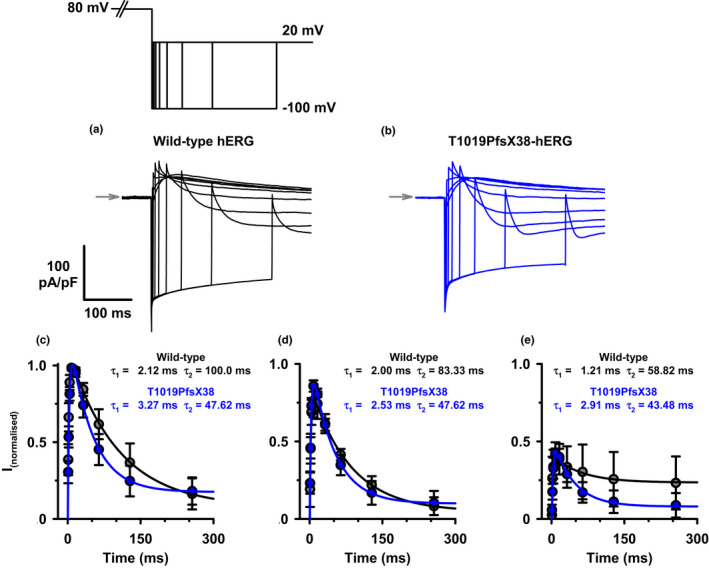
The time dependence of WT‐ and T1019PfsX38‐hERG recovery from inactivation and deactivation. (a, b) Representative whole‐cell currents recorded from WT‐ (a) and T1019PfsX38‐hERG (b) channels expressed in HEK293 cells. The currents were elicited using the protocol shown above A (V_hold_ = −80 mV). The example recordings in (a) and (b) were from cells activated at 80 mV. The grey arrows mark the level where V_C_ was 80 mV. (c–e) Quantification of the negative peak tail currents obtained after 80 mV (c) 40 mV (d) and 0 mV (e) test potentials. The values in (c) to (e) are shown after normalization, the normalization range was from the lowest peak current amplitudes (at 0 mV) to the highest peak current amplitudes (at 80 mV) in the same cell. The continuous lines in (c–e) are best fits to the average data using a 2‐component exponential function, τ_1_ is the time constant for recovery from inactivation and τ_2_ is the time constant for deactivation. Data are mean ± SEM, WT‐hERG, *n* = 4, grey circles; T1019PfsX38‐hERG, *n* = 4, blue circles. Other details as in Materials and Methods and Results sections.

To isolate the process of recovery from inactivation from that of deactivation and to determine the effects of the preceding test potential on these parameters, we utilized a protocol that measures the extent and rate of recovery from inactivation at repolarizing potentials. WT‐ and T1019PfsX38‐hERG expressing cells were depolarized for 2 s to either 80, 40 or 0 mV. Then the cells were stepped to −100 mV for different durations before depolarizing the membrane again to 20 mV. Then, peak tail currents were measured at 20 mV and plotted against the recovery time durations. Figure 6a,b show example recordings obtained using this protocol at 80 mV test potential. The quantifications of peak tail currents are presented in Figure 6c,e. The channels almost fully recovered from inactivation around 10 ms after stepping to −100 mV. There were no apparent differences in the rates of recovery from inactivation between WT‐ and T1019PfsX38‐hERG. Consistent with data in Figure 5b,c, the deactivation was faster for T1019PfsX38‐hERG when compared with WT‐hERG (Figure 6c‐e). The time constants of WT‐hERG deactivation at −100 mV positively correlated with the proceeding activation potential (Figure 6c‐e). In contrast, there was no correlation between the activation potentials and the deactivation time constants of T1019PfsX38‐hERG (Figure 6c‐e).

### 3.6 The voltage dependence of inactivation

**FIGURE 7:**
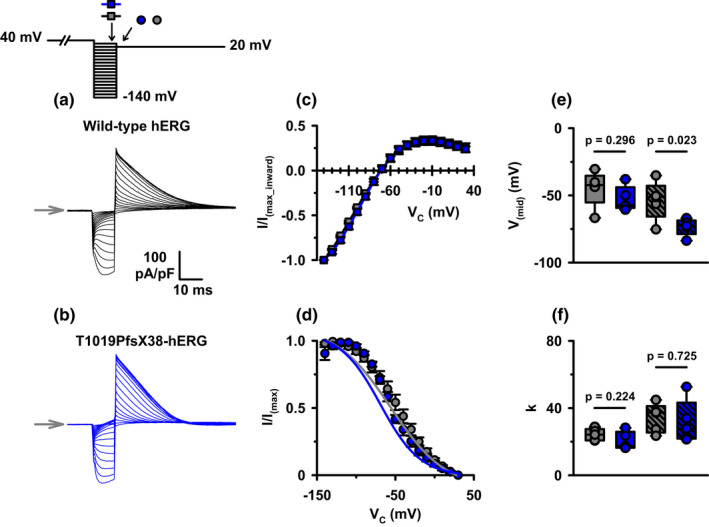
The voltage dependence of inactivation of WT‐ and T1019PfsX38‐hERG channels. (a, b) Representative whole‐cell currents recorded from WT‐ (a) and T1019PfsX38‐hERG (b) channels expressed in HEK293 cells. The currents were elicited using the protocol shown above A (V_hold_ = −80 mV). The grey arrows mark the level where V_C_ was 40 mV. (c) Quantification of currents measured at 1 ms before the end of the test potential and normalized to the maximal inward peak current amplitude. (d) Quantification of normalized tail currents measured at 5 ms after stepping to 20 mV. The grey and blue continuous lines in d are best fits to the average data after correction for deactivation. (e, f) Whisker plots of the V_mid_ and the k values as predicted by fitting the raw (open boxes) and the corrected (dashed boxes) data in d. Data are mean ± SEM; WT‐hERG, *n* = 5, grey boxes, squares, circles, and lines; T1019PfsX38‐hERG, *n* = 5–6, blue boxes, squares, circles, and lines. Other details as in Materials and Methods and Results sections.

hERG is a strong inward rectifier due to the rapid inactivation kinetics of the channel at depolarizing potentials (Smith et al., 1996). To investigate whether T1019PfsX38 affects the voltage dependence of inactivation, we utilized a voltage clamp protocol to induce recovery from inactivation followed by instantaneous depolarization to approximate the relative amount of reopened channels at the tested potentials. Figure 7 shows the protocol that we used to investigate the voltage dependence of inactivation of WT‐ and T1019PfsX38‐hERG. Briefly, the cells were depolarized for 4 s to 40 mV and then stepped to different test potentials for 10 ms. The relative amount of reopened channels were estimated by a subsequent step to 20 mV. Figure 7a,b show representative recordings of the currents that were obtained using this protocol. Visual inspection of the example recordings demonstrates that both WT‐ and T1019PfsX38‐hERG channels recovered from inactivation shortly after repolarization. Stepping to 20 mV induced instantaneous outward currents that decayed to reach a steady‐state level within <100 ms. Figure 7c presents quantifications of the normalized currents at the end of the 10 ms recovery step plotted against the tested potentials. Both variants had similar curves of normalized currents at the end of the recovery steps (Figure 7c). Figure 7d shows quantifications of the normalized tail currents at 20 mV plotted against the preceding test potentials. The IV relationship of WT‐hERG inactivation had an inverted sigmoidal correlation (Figure 7d). In contrast, the shapes of the inactivation IV curves of T1019PfsX38‐hERG were different from WT‐hERG curves (Figure 7d). The currents from the mutant variant appeared as if they inwardly rectify at hyperpolarizing potentials (Figure 7d). We attributed this to the faster rates of deactivation of T1019PfsX38‐hERG at hyperpolarising potentials when compared with WT‐hERG (Figure 5b,c). To correct for this, we approximated the expected amplitudes of currents that would have deactivated at the end of the 10 ms test potentials in Figure 7 (Vandenberg et al., 2012). We used the average values of the deactivation rate constants (Figure 5b,c) and the fractional amplitudes (Table 1) to approximate the amount of the deactivated currents after 10 ms of step potentials. For comparison, Figure 7e,f show the values of the V_mid_ and k of inactivation before and after correction. There were no apparent differences in V_mid_ and k of inactivation when the curves were fitted before correction (Figure 7e‐f). After correction for deactivation at hyperpolarised potentials, the values of the V_mid_ of T1019PfsX38‐hERG were shifted by ~20 mV in the negative direction when compared with WT‐hERG (Figure 7e). There were no statistically significant differences in the values of k between the two variants after correction (Figure 7f).

### 3.7 The rates of hERG channel inactivation

**FIGURE 8:**
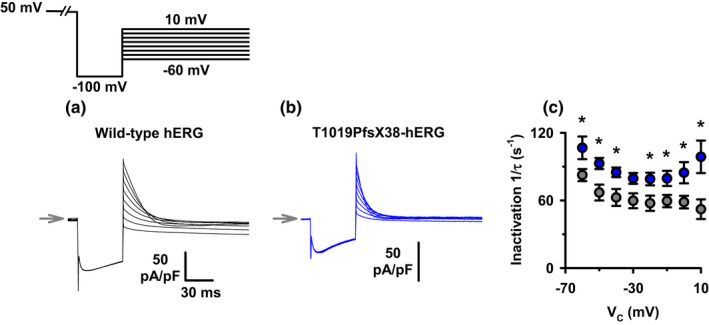
The inactivation rates of WT‐ and T1019PfsX38‐hERG channels. (a, b) Representative whole‐cell currents recorded from WT‐ (a) and T1019PfsX38‐hERG (b) channels expressed in HEK293 cells. The currents were elicited using the protocol shown above A (V_hold_ = −80 mV). The grey arrows mark the level where V_C_ was 50 mV. (c) Quantification of the rate constants of channel inactivation measured by fitting the tail currents at the different tested potentials using a standard exponential function. Data are mean ± SEM, *p < 0.05; WT‐hERG, *n* = 7 except at −10 mV (*n* = 6), grey circles; T1019PfsX38‐hERG, *n* = 5, blue circles. Other details as in Materials and Methods and Results sections.

To test whether the T1019PfsX38 variant of hERG affects channel inactivation rates, we utilized a protocol to force the reopened channels to instantaneously re‐inactivate at different depolarizing test potentials. Figure 8 shows a protocol which we utilized to measure the inactivation rates of WT and mutant channels. Briefly, the cells were depolarized to 50 mV for 2 s, then hyperpolarized to −100 mV for 50 ms. The reopened channels during hyperpolarization were forced to inactivate by immediately stepping the membrane potential to different depolarizing voltages. Figure 8a,b show example recordings using this protocol on cells expressing WT or mutant hERG channels. Visual inspections of both example recordings indicate that the reopened channels at −100 mV produced hooked tails which decayed almost completely within less than 50 ms following depolarization. T1019PfsX38‐hERG had faster rates of channel inactivation than WT‐hERG at all tested potentials. Figure 8c shows quantification of the rate constants of channel inactivation at different depolarizing potentials. T1019PfsX38 increased the rates of hERG channel inactivation by 20–26 s^−1^ at most tested potentials (Figure 8c). Thus, we conclude that T1019PfsX38 accelerates hERG channel inactivation rates at depolarized potentials.

### 3.8 The response of WT‐ and T1019PfsX38‐hERG to voltage step‐ramp protocol

**FIGURE 9:**
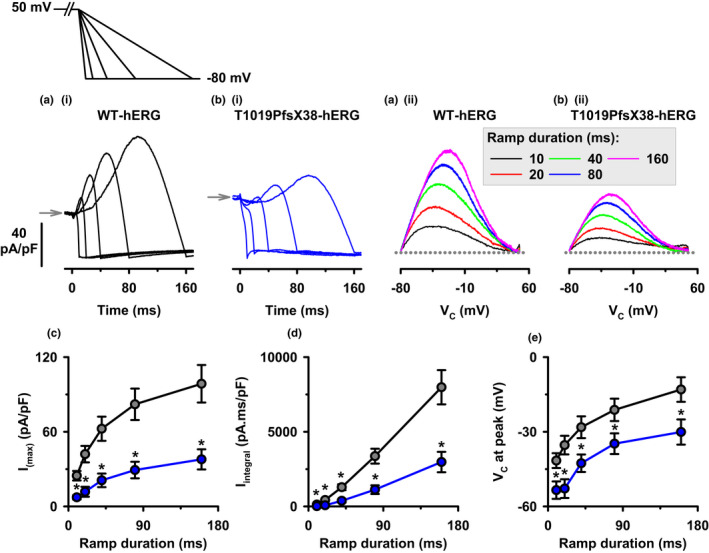
Step‐ramp voltage clamp protocol on cells expressing WT‐ or T1019PfsX38‐hERG. (ai and bi) Example current recordings from HEK293 cells that were transfected with WT‐ (ai) or T1019PfsX38‐hERG (bi) channels, as indicated. The currents were elicited using the protocol shown above A (V_hold_ = –80 mV). The grey arrows mark the level where V_C_ was 50 mV. (aii and bii) Current recordings from (ai) and (bi) presented as functions of membrane voltage. The ramp durations in (aii) and (bii) were colour‐coded as indicated in the box. The dotted lines represent ramp slope lines between 50 and –80 mV. (c) Average densities of peak current amplitudes plotted as functions of ramp durations. (d) Average values of area under each curve of current densities (I_integral_) at different ramp durations. (e) Average values of membrane voltages at the time of occurance of peak current amplitudes. Data are mean ± SEM, *p < 0.05; WT‐hERG, *n* = 12, grey circles; T1019PfsX38‐hERG, *n* = 11 (except in E where *n* = 7 at 10 ms ramp duration), blue circles. At 20 ms in d and 40 ms in c and d the individual data points obtained from T1019PfsX38‐hERG did not meet normal statistical distribution criteria (Shapiro–Wilk test), in which case Mann–Whitney rank sum test was used for statistical comparison only at these three data points. Other details as in Materials and Methods and Results sections.

The differences seen between WT‐ and T1019PfsX38‐hERG functions at steady‐state conditions (Figures 3, 4, 5, 6, 7, 8) provide important but incomplete insight into the behaviors of these channels under different conditions. To better decipher their behaviors at quasi‐physiological conditions, we employed a voltage step‐ramp protocol to maximally activate hERG channels before repolarizing the membrane gradually to the resting membrane potential. Briefly, we stepped V_m_ from a holding potential of –80 to 50 mV for 2 s then gradually ramped V_m_ to −80 mV using various ramp speeds. Figure 9 presents example recordings and quantification of WT‐ and T1019PfsX38‐hERG currents and their characteristics obtained using the different voltage ramp durations. Visual inspection of the example recordings in Figure 9ai‐bi indicates that T1019PfsX38‐hERG expressed in HEK293 cells produced lower amplitudes of currents than WT‐hERG. The recordings indicate that WT‐ and T1019PfsX38‐hERG activities increased to maximal peaks while the membrane was repolarizing, then decreased until reaching resting states. The increase in current during repolarization was slower in T1019PfsX38‐hERG than WT‐hERG expressing cells (Figure 9ai‐bi). Figure 9aii,bii present the former recordings as functions of membrane voltage, assuming linear slopes of leak currents between 50 and −80 mV. WT‐ and T1019PfsX38‐hERG expressing cells produced bell shaped outward current curves that peaked between 0 and −60 mV (Figure 9aii,bii). To better characterize the behavior of these channels during voltage ramps, we measured the amplitudes of peak currents and the area under each curve and presented the resulting average data in Figure 9c,d, respectively. T1019PfsX38‐hERG was associated with smaller peak currents (I_(max)_, Figure 9c) and current integrals (I_integral_, Figure 9d) than WT‐hERG at all tested ramp durations (*p* < 0.05). The voltage at peak currents during membrane repolarization varied depending on the repolarization speed. Figure 9e presents quantification of the membrane potentials at peak currents. Peak currents through WT‐ and T1019PfsX38‐hERG occurred at negative potentials (Figure 9e). However, the peaks of WT‐hERG currents were reached earlier during repolarization when compared with the peaks of T1019PfsX38‐hERG currents (Figure 9e). The peaks of T1019PfsX38‐hERG currents were delayed by 10.8%–19.7% compared with the time of the peaks of WT‐hERG currents during membrane repolarization. This translates to 12–17.5 mV differences in membrane voltage at peak current amplitudes between WT‐ and T1019PfsX38‐hERG expressing cells (*p* < 0.04) (Figure 9e). Notably, larger differences can be observed between WT‐ and T1019PfsX38‐hERG during longer voltage ramp durations (Figure 9c‐e).

## 4 DISCUSSION

This study investigated the functional characteristics T1019PfsX38‐hERG expressed in HEK293 cells. This variant modifies the largely disordered distal region of hERG. We demonstrated that T1019PfsX38 modifies the deactivation kinetics of hERG and alters the apparent voltage dependence of inactivation. Interpretation of the data suggests that changes in the gating kinetics would contribute to the pathophysiology of T1019PfsX38 when the channel is at the plasma membrane. To provide an insight, we conducted step‐ramp stimulations on WT‐ and T1019PfsX38‐hERG expressing cells. T1019PfsX38‐hERG expression achieved smaller densities of K^+^ currents than WT‐hERG when stimulated using the step‐ramp protocol. We believe that this is primarily caused by changes in the channel gating dynamics. This observation interprets the prolonged QTc phenotype seen in a homozygote T1019PfsX38‐hERG patient reported by Al Senaidi et al. (2014).

Clinically, the homozygote patient described by Al Senaidi et al. (2014) carried the T1019PfsX38‐hERG variant and had a markedly prolonged QTc interval of >570 ms. In addition, she had incomplete right bundle branch block and presented with dilatation of the ascending aorta and pulmonary artery, and left ventricular non‐compaction. Our data predicts that the mutant variant has reduced activity when studied using dynamic voltage clamp conditions (Figure 9). This pathology can be an important cause for the prolonged QTc in the reported proband. However, full genotype‐to‐phenotype correlations in carriers of the mutation require additional genetic and functional evidence.

About 88% of the reported LQT2 mutations cause trafficking defects in the hERG channel (Smith et al., 2016). The cDNA of T1019PfsX38‐hERG had comparatively similar functional expression to WT‐hERG in HEK293 cells (Figure 2). Based on our data T1019PfsX38‐hERG can be classified as having Class 3 defects. It is not clear whether other classes of channel defects might contribute to the molecular pathophysiology of T1019PfsX38. For example, nonsense‐mediated mRNA decay (NMD) reduces the expression levels of many frameshift and nonsense mutations by degrading hERG mRNA (Gong et al., 2014). Whether NMD contributes to the pathogenesis of T1019PfsX38‐hERG remains to be determined and could only be studied using intron containing DNA constructs (Gong et al., 2014).

Many data exists about the structural contribution of the different protein domains to hERG channel function. Previous studies demonstrated the involvement of the N‐ and C‐termini in channel gating (Aydar & Palmer, 2001; Gustina & Trudeau, 2011). Certain deletions in both ends of the channel accelerate the rates of channel deactivation at hyperpolarized potentials (Aydar & Palmer, 2001). Inter‐subunit interactions between both termini have been demonstrated using functional and structural data (Gianulis et al., 2013; Wang & MacKinnon, 2017). The C‐terminus physically interacts with the N‐terminus of the adjacent subunit in the tetrameric structure of hERG. This is influenced by an interface which forms between the cNBHD and the eag region. These interactions stabilize the open configuration of the channel (Gianulis et al., 2013; Muskett et al., 2011). Although the C‐tail is structurally distinct from the cNBHD (Figure 1), modification of the C‐tail by T1019PfsX38 destabilizes the open configuration of hERG and accelerates the inactivation and deactivation rates (Figures 5 and 8).

The involvement of the C‐tail in channel gating was observed in a few mutagenesis studies, including those conducted on frameshift variants. Truncations at the C‐terminus had various effects on channel activation and deactivation kinetics (Aydar & Palmer, 2001; Bhuiyan et al., 2008). P1122PfsX148 (legacy name: 1122fs/147) increased the rates of hERG inactivation and shifted its voltage dependence 11 mV in the negative direction (Sasano et al., 2004). The C‐tail also appears to be the site of interaction with proteins that modify the gating kinetics of hERG. The beta‐adrenergic modification of hERG was found to be absent in the R1014PfsX39 and V1038AfsX21 variants due to the loss of key binding sites of the adaptor protein 14–3‐3epsilon (Choe et al., 2006). It is likely that interactions between hERG and other proteins are affected by changes introduced by T1019PfsX38.

Slow deactivation is a distinguishing feature of hERG channel gating. Acceleration of the deactivation rates would reduce the open probability of hERG at the end of the cardiac AP, which can prolong the AP duration. In our hands, T1019PfsX38 causes faster deactivation rates at hyperpolarized, but not depolarized potentials (Figure 5). The cardiac AP is largely depolarizing. Whether the accelerated deactivation rates contributed to the lower outward currents in the step‐ramp protocol of T1019PfsX38‐hERG expressing cells (Figure 9) requires further analyses. The movement of the voltage sensor domain (VSD) is a rate‐limiting process in deactivation, influenced by the connections that it forms with other residues and by intrinsic mechanisms. The VSD of K_V_ channels has three major conformations, one “down” and two “up” positions (Villalba‐Galea et al., 2008). Upon repolarization, the VSD moves from the down position to a preliminary up position. Then, it forms interactions that stabilize its positon in the up conformation. The stability of the second up configuration depends on the strength of the formed interactions, which are in turn dependent on the duration of the depolarization step (Thouta et al., 2017). The S4–S5 linker and the N‐terminus are modulators of the rates of VSD relaxation upon repolarization. The S4–S5 loop links the pore domain with the VSD and mediates the communication between these structures during the process of deactivation (Thouta et al., 2017). The N‐terminus, on the other hand, stabilizes both of the VSD and the pore domain upon repolarization (Thouta et al., 2017). Our results and others’ demonstrate the involvement of the C‐terminus in shaping the deactivation rates (Figure 5) (Aydar & Palmer, 2001; Gustina & Trudeau, 2011). We found that T1019PfsX38 affects the fast and the slow deactivation rates (Figure 5) without affecting the relative contribution of these components to the process of current relaxation (Table 1). Due to the position of the C‐tail and its largely disordered shape (Figure 1), we think that the C‐tail regulates hERG channel deactivation by structurally different mechanisms when compared with the S4–S5 linker and the N‐terminus and it might involve allosteric modulation of conformational waves that travel along the transmembrane segments. It might be logical to think that T1019PfsX38‐hERG lowers the height of an energy barrier that slows the transition of hERG from the active to the closed states at hyperpolarising membrane potentials.

The inactivation process of hERG resembles C‐type mechanisms  which stop ion flow through the selectivity filter (Cordero‐Morales et al., 2006; Schönherr & Heinemann, 1996; Smith et al., 1996). The transition between the open and the inactive states involves widespread transmembrane and intracellular motions which lead eventually to pore closure by C‐type rearrangements (Wang et al., 2010). The inactivation process is conserved in T1019PfsX38‐hERG, despite differences in the sensitivities to membrane voltage in comparison with WT‐hERG (Figures 4, 7, and 8). It is difficult to determine the exact mechanisms by which this variant changes the voltage sensitivity of inactivation. The negative shift in the voltage dependence of inactivation by T1019PfsX38 (Figure 7) can be a result of lowering the energy barrier that slows the transition from the open to the inactive states at depolarizing conditions. This is also manifested as accelerated rates of inactivation of T1019PfsX38‐hERG channels (Figure 8). The strong inward rectification of the mutant channels at depolarizing conditions (Figure 4) and the negative shift in the voltage dependence of inactivation (Figure 7) elucidate the mechanisms by which T1019PfsX38 reduces the outward currents during repolarization (Figure 9). WT‐hERG channels start recovering from inactivation earlier during repolarization when compared with T1019PfsX38‐hERG (Figures 7 and 9). During membrane repolarization, K^+^ ions passing through reopened WT‐hERG experience stronger electrical driving force than ions passing through T1019PfsX38‐hERG. This explains the differences in the current amplitudes between WT‐ and T1019PfsX38‐hERG  seen in Figure 9.

Overall, our results provide mechanistic insights into the behavior of T1019PfsX38‐hERG when expressed on the plasma membrane of a human cell line. However, additional genetic and functional investigations might be required to understand the correlation between genotype and phenotype in the reported mutation carriers, including explanation for the presence of incomplete right bundle branch block, dilatation of the ascending aorta and pulmonary artery, and left ventricular non‐compaction (Al Senaidi et al., 2014). Better functional studies using the patch‐clamp technique could be performed at 37°C (Lei et al., 2019; Vandenberg et al., 2006).

## 5 CONCLUSION

We provided evidence for the classification of the T1019PfsX38 LQT2 mutation as a class 3 variant. The modification of the distal tail by this variant accelerated the rates of channel deactivation at hyperpolarized potentials, and the rates of channel inactivation at depolarized potentials, both reflecting instability of the open state in the mutant channel. The structural interplay between the intracellular and the transmembrane regions of hERG requires additional attention, including those that involve the distal C‐terminal tail region. In summary, changes in the hERG channel gating dynamics by T1019PfsX38 might reduce I_Kr_ currents in native cardiac myocytes.

## AUTHORS CONTRIBUTIONS

Conception and design of the experiments: Majid K. Al Salmani; performed the research: Majid K. Al Salmani, Rezvan Tavakol, and Wajid Zaman; analysis and interpretation of data: Majid K. Al Salmani; drafting the article: Majid K. Al Salmani; revising the article critically for important intellectual content: Majid K. Al Salmani and Ahmed Al Harrasi. All authors approved the final version of the manuscript. All experiments were performed at the University of Nizwa, Nizwa, Oman.

## CONFLICT OF INTEREST

The authors have no conflict of interest to declare.
